# Açai Seeds (*Euterpe oleracea* Mart) Are Agroindustrial Waste with High Potential to Produce Low-Cost Substrates after Acid Hydrolysis

**DOI:** 10.3390/molecules28186661

**Published:** 2023-09-16

**Authors:** Willen Silva Igreja, Luiza Helena da Silva Martins, Rafaela Rodrigues de Almeida, Johnatt Allan Rocha de Oliveira, Alessandra Santos Lopes, Renan Campos Chisté

**Affiliations:** 1 Postgraduate Program of Food Science and Technology (PPGCTA), Institute of Technology (ITEC), Federal University of Pará (UFPA), Belém 66075-110, PA, Brazil; 2 Instituto de Saúde e Produção Animal (ISPA), Universidade Federal Rural da Amazônia (UFRA), Belém 66075-900, PA, Brazil; 3 School of Food Engineering (FEA), Institute of Technology (ITEC), Federal University of Pará (UFPA), Belém 66075-110, PA, Brazil; 4 Nutrition College, Federal University of Pará (UFPA), Belém 66075-110, PA, Brazil

**Keywords:** Amazonian fruits, byproduct, reducing sugars, fiber seeds, bioeconomy

## Abstract

Açai seeds have been discarded improperly around the Amazonia region, but they can be seen as promising low-cost substrates for fermentation processes. The structural carbohydrates and physicochemical characterization of açai seeds from the Amazonia were assessed followed by the determination of the optimal hydrolysis conditions using H_3_PO_4_ (phosphoric acid) and H_2_SO_4_ (sulfuric acid) to obtain a liquor with high contents of simple carbohydrates and low levels of potential microbial inhibitors usually generated during acid hydrolysis of carbohydrates. A central composite rotational design was carried out varying the concentrations of diluted acid (0–5%, *w*/*v*), solids (0.1–25%, *w*/*v*), and hydrolysis time (9.5–110 min). Acid hydrolysis with H_2_SO_4_ was more effective in producing reducing sugars (15.9–103.1 g/L) than H_3_PO_4_ (2.9–33.9 g/L) during optimization. The optimal hydrolysis conditions with H_2_SO_4_ were 3.5% of acid (*w*/*v*), 25% of solids during 70 min at 121 °C, which provided a liquor with 55 g/L of reducing sugars and low levels of microbial inhibitors: acetic acid (1.8 g/L), hydroxymethyl furfural (338 mg/L), and furfural (10 mg/L). Thus, açai seeds were characterized as promising agroindustrial waste with high potential to be used as a low-cost substrate in biotechnological processes, comprising relevant environmental and bioeconomic aspects for the development of the Amazonia.

## 1. Introduction

The world economy has improved in production systems with increasingly higher productivity yields and lower operating costs that provide increasingly profitable agroindustrial production. In line with environmental and bioeconomic aspects, it is of paramount importance that raw materials, and derived wastes, be used in any technological processes in its entirety. In this sense, the Amazon highlights its contribution in the agroindustrial market with açai fruits being one of the main exploited products, yielding more than 540 million USD in 2019 [1].

Despite the expressive numbers, during açai processing, to obtain the juice, only 15% of the fruit is used in the process (epicarp and mesocarp), with the remaining 85% composed by fibers (endocarp), which are improperly discarded around Amazonia [2]. Waste with fibrous characteristics formed by cellulose, lignin, and hemicellulose are susceptible to pre-treatments to release fermentable sugars [3], which can further be used as a culture media in biotechnological processes, with the aim of promoting and produce high value-added products from low-cost substrates, providing cost savings in the process.

The study of the use of new industrial fermentation means from agribusiness waste has been intensified in recent years [3,4], and it can be produced from various lignocellulose complexes representing a cheap source, renewable and plentiful, enabling sustainable and profitable production. In view of this, several pre-treatments have been reported in the literature with the aim of fractionating, solubilizing, hydrolyzing, and separating the biomass components for adequate purposes, with acid pre-treatment being one of the most promising in deconstructing the lignocellulose chain and changing the conformation structure of lignins [5].

At the moment, there is a scarcity of data in the literature that assess the potential application of agroindustrial wastes, namely açai seeds, as a substrate for the production of high-added value products. Therefore, the objective of this work was to investigate the acid hydrolysis treatment of açai seeds to produce a liquor with high contents of simple carbohydrates and low levels of potential microbial inhibitors usually generated during acid hydrolysis of carbohydrates, which were monitored for the first time, for the application as low-cost substrate in further biotechnological processes. The innovative nature of this work is that it provides a potential socioeconomic and environmental alternative through the waste generated by the açai production chain, making it possible to cheapen several fermentable processes to produce economically viable products.

## 2. Results and Discussion

### 2.1. Characterization of the Dried Açai Seeds

**Figure 1:**
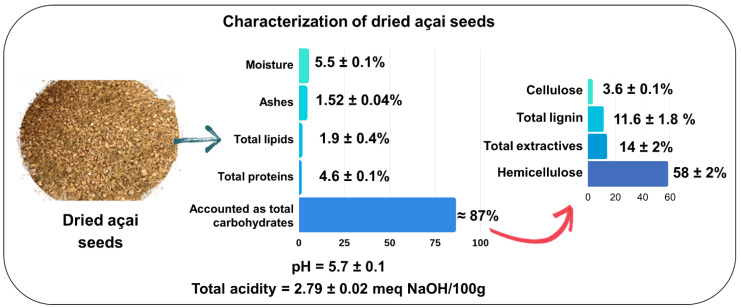
Physicochemical and structural carbohydrates characterization of the dried açai seeds used in this work.

Açai seeds are agroindustrial wastes that are still little explored, with few scientific data available in the literature, and their physicochemical composition is essential to enable their application in a variety of investigations, including as a substrate in biotechnological approaches. As can be seen in Figure 1, the major constituent of the dried seeds was accounted for as total carbohydrates (≈87%), and according to the literature [6,7,8,9], due to the high content of lignocellulosic material, açai seeds can be considered a promising low-cost substrate for the production of derived compounds with high added value by biotechnological means.

However, the determination of total carbohydrates, by difference, overestimated the total carbohydrate values in the dried açai seeds since it also included the contents of total extractives and total lignin. Thus, after determining the contents of total extractives, structural carbohydrates, and total lignin, the dried açai seeds exhibited low contents of cellulose (3.6%) and total lignin (11.68%), 14% of total extractives and high hemicellulose contents (58%) (Figure 1). According to Oliveira et al. [6], the fibers of *E. oleracea* seeds presented 38% cellulose, 18% lignin, and 25% hemicellulose; and in another study, Martins et al. [10] reported that the fibers of the açai seeds were composed of 37% hemicellulose, 33% lignin, and 33% cellulose. A recent study showed that the fibers of açai seeds are mainly composed of hemicellulose (50%, dry basis) [11], which supported our findings. Based on these values, açai seeds presented a differentiated composition of lignocellulosic material to be used directly as a substrate for fermentation processes, requiring pre-treatment strategies such as acid hydrolysis to release simple fermentable sugars.

In relation to the other constituents (Figure 1), the moisture content of the dried açai seeds (5.5%) was lower than those found in other studies with dehydrated açai seeds (8 and 13%) [6,7], but it clearly depends on the chosen drying parameters. Importantly, when a dried material is used for the purpose of releasing fermentable sugars through acid hydrolysis, moisture becomes a preponderant factor, as high values may dilute the acid concentration, decreasing the hydrolysis efficiency, and values < 10% are the most applicable [12,13]. The total protein values found in this work (4.6%) were similar to those found in other studies (1.7–4.3%) [6,7,8,9], and the total lipid values (1.9%) were similar to those found by Ferreira et al. (1.70%) [8]. Additionally, based on the pH and total acidity values (Figure 1), the dried açai seeds used in this study can be considered a low acidic product. In general, the variations in the contents of moisture, total proteins, total lipids, and ashes in the seeds are usually attributed to differences in planting conditions, ripening stage, climate, geographic location, harvest period, time of sun exposure, and its own genetic variability.

### 2.2. Effect of the Acid Hydrolysis Treatments on the Dried Açai Seeds

During acid hydrolysis with diluted acid, the lignocellulosic material is hydrolyzed causing the release of sugar monomers [14]. Although H_3_PO_4_ has a higher cost than other acids, such as H_2_SO_4_ and hydrochloric (HCl), it is considered a weaker acid (and consequently, lower combined severity factor) as compared to the previously mentioned acids, and its use implies lower environmental impact, in addition to being less corrosive and toxic. Moreover, H_3_PO_4_ has phosphorus in its composition, which can be incorporated as a micronutrient by microorganisms after hydrolysis [15,16].

In our study, although H_3_PO_4_ presented lower combined severity factors than H_2_SO_4_ for the selected acid hydrolysis conditions in the central composite rotational design (CCRD) runs, the range of total reducing sugar contents in the produced liquor (2.93–33.97 g/L) (Supplementary Table S1) was much lower than the contents obtained by H_2_SO_4_ (6.93–103.12 g/L) (Table 1). Thus, we decided to focus our investigation on only the H_2_SO_4_ treatment, but all the results for the acid hydrolysis with H_3_PO_4_ can also be found as Supplementary Material.

According to Table 1, in general, the contents of total reducing sugars increased as the combined severity factor increased during the acid hydrolysis of dried açai seeds, and the same tendency was observed for H_3_PO_4_ (Supplementary Table S1). For H_2_SO_4_, the increase in the acid concentration from 0 (run 9) to 2.5% (runs 15, 16, and 17) increased by about seven times the contents of total reducing sugars. If the acid treatments are carried out under mild conditions of acid concentration and temperature, the hemicellulose fraction may be extracted without significantly affecting cellulose and lignin contents [14]. Furthermore, hydrolysis time also has relevant effects to be considered since the increase in time from 60 (run 12) to 110 min (runs 15, 16, and 17), at the same acid and solid concentrations, did not increase the contents of reducing sugars in the liquor. This behavior can be explained due to the higher severity of the treatment at 110 min than 60 min that probably degraded sugars resulting in the formation of derived products [5,17]. Thus, monitoring the combined severity factor and time during acid hydrolysis may ensure the release of glycoside monomers while avoiding or minimizing the accumulation of sugar degradation products, such as hydroxymethylfurfural (HMF) [5].

The highest content of total reducing sugar in the liquor after the H_2_SO_4_ treatments (103.12 g/L) was obtained at 4% acid and 20% solids after 90 min of hydrolysis (Table 1). Interestingly, these same conditions also yielded the highest contents of total reducing sugars to the liquor after the hydrolysis using H_3_PO_4_ (33.97 g/L) (Supplementary Table S1).

**Figure 2:**
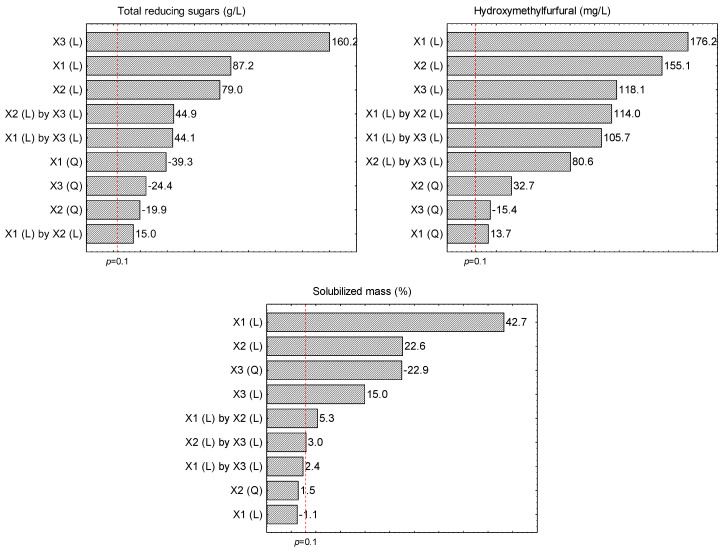
Pareto charts for the standardized effect estimates (absolute values) on the contents of total reducing sugars, hydroxymethylfurfural, and solubilized mass after the acid hydrolysis of dried açai seeds using H_2_SO_4_. X1 = Acid concentration (%, *w*/*v*); X2 = Hydrolysis time (min); X3 = Solids concentration (%, *w*/*v*); (L): linear effect; (Q) quadratic effect.

According to the Pareto charts (Figure 2), the contents of solids and acid concentration were the variables that most affected (*p* ≤ 0.1) the contents of total reducing sugars, with positive linear effects, followed by the hydrolysis time, suggesting that the higher the percentage of dried açai seeds and the concentration of H_2_SO_4_, the higher the total reducing sugar contents in the liquor. Furthermore, the treatments with H_2_SO_4_ resulted in a very low variation in the contents of total reducing sugars at the CCRD central points (relative standard deviation (RSD) = 1%), which indicated high repeatability of the hydrolysis procedure. Statistical details regarding the values of the *t* and *p* tests carried out for the experimental design for all the parameters observed in this study can be found as Supplementary Table S2.

As the acid treatment acts mainly in the solubilization of hemicelluloses, very severe treatments degrade sugars during hydrolysis, generating microbiological inhibitors, such as HMF, which may affect fermentation processes [5]. As can be seen in Table 1, the increase in the combined severity factor contributed to the increase in the HMF contents in the hydrolyzed liquor, with the highest concentration found in run 8 (448 mg/L), which corresponds to the highest factor (1.68). Oliveira et al. [6] carried out pre-treatment of açai seeds using diluted H_2_SO_4_ under various experimental conditions of acid concentration (0.5 to 1% *m*/*v*), hydrolysis time (9.5 to 111 min), and solids concentration (1.6 to 18%), and reported HMF contents ranging from 1 to 221 mg/L. The milder combined severity factor and lower solids concentration range in comparison with our study may explain the lower contents of HMF. HMF can cause damage to yeast cells as the presence of the hydroxymethyl group reduces both the hydrophobicity and membrane permeability, resulting in reduced rates of HMF conversion [18]. These same authors stated that in the presence of HMF, fermentative microorganisms redirect the energy of the cells to repair the damage, resulting in a reduction in intracellular levels of ATP and NADPH caused by enzymatic inhibition or by consumption/regeneration of cofactors.

The Pareto charts for the contents of HMF (Figure 2) showed that acid concentration and hydrolysis time were the variables that most influenced (*p* ≤ 0.1) its contents, with linear positive effects, followed by the percentage of solids, suggesting that the higher the concentration of H_2_SO_4_ and hydrolysis time, the higher the HMF contents in the liquor. These results were expected, as the use of high concentrations of acid, combined with high temperatures, promotes the dehydration of hexoses and consequently the production of furans [5,17].

Regarding the contents of solubilized mass of the solid fractions of the dried açai seeds after the acid hydrolysis with H_2_SO_4_, the lowest percentage (11.56%) was observed at 0% of diluted acid, and the mass solubilization increased in the runs with acid addition (1–5%) from 21.53 to 49.58% (Table 2). According to the Pareto charts (Figure 2), the positive linear effect of acid concentration was the variable that most affected (*p* ≤ 0.1) the mass solubilization, followed by the negative quadratic effect of solids percentage and linear positive effect of hydrolysis time, suggesting that the solid fraction of the dried açai seeds was more soluble at the highest tested H_2_SO_4_ concentration and hydrolysis time. According to the literature, the acid pre-treatment of most lignocellulosic materials results in high solubilization percentages of hemicellulose and a partial digestion of cellulose leading the formation of monomer or oligomer sugars [15,16,19].

**Figure 3:**
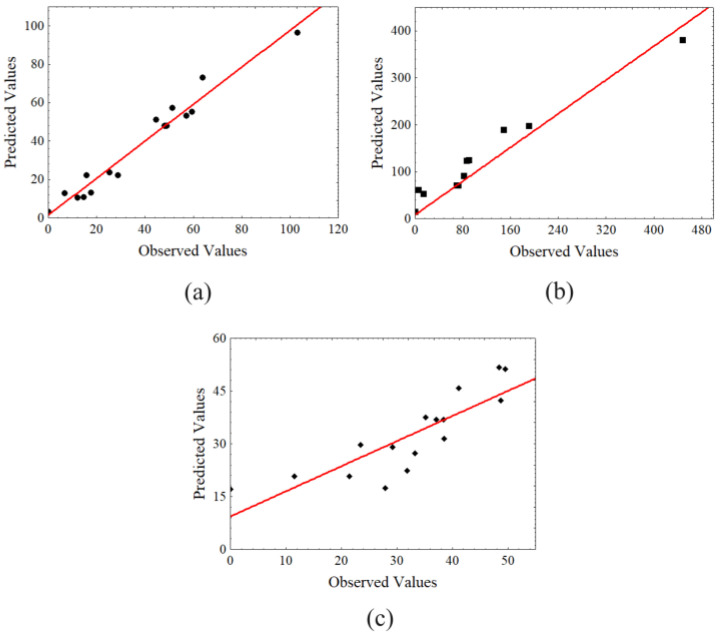
Graph of the observed and predicted values of the experimental design with central composite and axial levels of the studied parameters (**a**) TRS, (**b**) HMF, and (**c**) solubilized mass.

Figure 3 shows the behavior reflected in the distribution of residues, where the data from the present study are arranged randomly and not with a curved pattern for the solubilized mass (Figure 3c). Still, we no longer see the same behavior for TRS (Figure 3a) and HMF (Figure 3b), which indicates the need to adjust the mathematical model. In the calculated ANOVA (Supplementary Table S3), it was observed that there was a lack of fit for the data of this study; although the models studied were significant, they cannot be considered predictive; they can only be used as trend indicators, that is, only evaluated for the values of the range studied in the present work and cannot predict other values that may deviate from this range.

### 2.3. Optimal Conditions to Produce Liquor from Dried Açai Seeds by Acid Hydrolysis as Pre-Treatment Using H_2_SO_4_

After performing ANOVA (*p* ≤ 0.1), the second order polynomial models proposed by the response surface methodology were not adequate for the prediction of TRS, HMF, and SM contents, since they showed great lack of fit; they were significant but non predictive, despite their high and moderate R^2^ (0.96, 0.89, and 0.71, respectively) (Supplementary Table S3).

The regression models aim to establish a relationship that predicts the response corresponding to any independent variable value. For the test of the statistical significance of the regression, it was observed that the F distribution for TRS (20.11), with a 90% confidence interval, was greater than the F_table_ value (2.72). For HMF, the F_regression_ value (6.92), with a 90% confidence interval, is greater than the F_table_ value (2.72). Regression (1.93) was more significant for soluble solids than the F_table_ (2.72), which rules out the null hypothesis that there is no linear relationship between the dependent and independent variables.

**Figure 4:**
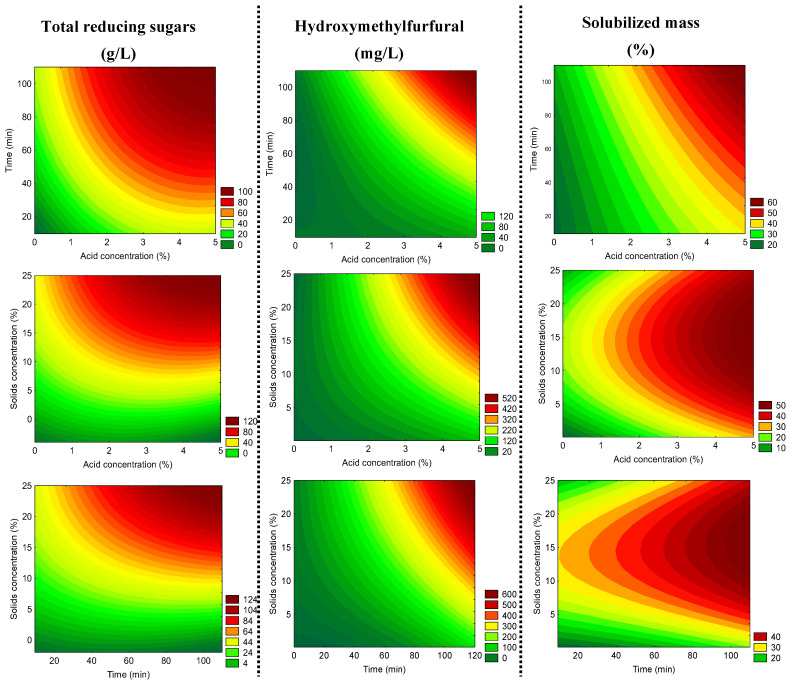
Contour plots to illustrate the contents of total reducing sugars (g/L), hydroxymethylfurfural (mg/L), and solubilized mass (%) for the determination of the optimal conditions for the acid hydrolysis of dried açai seeds using H_2_SO_4_.

However, a lack of fit was observed in all experiments, which may be due to the failure to fully evaluate the response surface, as can be seen in Figure 4 (for constructing the contour graph). Therefore, the complementation of the results obtained in the regression analysis was considered satisfactory for continuing studies in the range of our experimental design.

Concerning the formation of HMF after carbohydrate hydrolysis, the highest levels tend to be formed at longer times and both higher acid and solid concentrations, as expected. However, according to Figure 4, no relevant increase in the HMF contents is expected to be found as long as the acid concentration is kept below 2%, even at longer hydrolysis time and higher solids concentration. Lastly, in relation to the solubilized mass, the highest solubilization of lignocellulosic material in dried açai seeds will be achieved by the combination of the highest acid concentrations and hydrolysis time.

Considering that the liquor with the highest TRS content (103.12 g/L) was obtained at the highest H_2_SO_4_ and longer time, which also showed the highest HMF level, we decided to use the desirability function to determine the optimal acid hydrolysis conditions with reduced CSF that minimize the formation of HMF. Therefore, after considering all the tendencies shown by the contour plots (Figure 4), the desirability function was set to predict the contents of TRS, HMF, and solubilized mass at 3.5% (*w*/*v*) acid concentration (*m*/*v*), 70 min of hydrolysis time, and 25% (*w*/*v*) solids concentration (Supplementary Figure S1). The correct selection of the acid hydrolysis conditions should be carefully and systematically further investigated to provide industrially feasible results to establish an economical viable process; the higher the acid concentration, the higher the probability of reactors’ corrosion, increasing the maintenance price. Moreover, the need of high-power consumption due to long times as well as the need for additional costs for the neutralization of the acid liquor along with further purification steps must also be taken into account when designing any industrial project.

In order to validate the optimal acid hydrolysis conditions selected in our study for the dried açai seeds, we carried out the hydrolysis procedure (triplicate) and the produced liquor presented 82 ± 2 g/L of TRS, 265 ± 1 mg/L of HMF, and the solubilized mass was 41 ± 1%. The experimental values were close to the values predicted by the desirability function for TRS (94 g/L), HMF (273 mg/L), and solubilized mass (36%), with low relative standard deviation values (RSD = 9.6%, 2.1%, and 9.2%, respectively), demonstrating that the approached methodology is predictive and the conditions established for the acid hydrolysis procedure are reproductive.

### 2.4. Characterization of Sugars and Potential Microbial Inhibitors in the Newly Hydrolyzed Liquor and the Neutralized Liquor to Be Used as Substrate in Fermentation Processes

In our study, the newly hydrolyzed liquor obtained at the optimal acid hydrolysis conditions exhibited very low pH value (pH < 1.0) due to the use of 3.5% H_2_SO_4_, which characterizes it as a non-favorable substrate to be used for fermentation purposes, thus needing neutralization (pH close to 7.0). According to Table 2, the newly hydrolyzed liquor presented 55 g/L of sugars, mannose being the major monosaccharide (51 g/L) and accounting for about 93% of the detected sugars. After the neutralization of the liquor, the contents of mannose decreased by about 18% (42 g/L), and xylose by ≈26% (1.4 g/L), and were lower than those reported by Monteiro et al. [11] who found maximum values of 49.5 g/L for mannose in the liquor. For the contents of xylose, the values found hitherto varied from 1.9 to 1.4 (before and after neutralization) and were lower than the values observed by Oliveira et al. [6], which was 24.8 g/L, and by Monteiro et al. [11] with the maximum value of 3.26 g/L.

Several yeasts of the genus *Rhodotorula* spp., such as *R. glutinis*, *R. toruloides*, *R. graminis*, and *R. mucilaginosa*, have been investigated over the years as carotenoid producers by biotechnological means, and they were reported to be able to metabolize mannose and xylose as carbohydrate source [20,21,22,23,24], highlighting the high potential of açai seeds as low-cost substrates for the growth of selected microorganisms. As an example, Silva et al. [23] cultivated a strain of *R. glutinis* in a culture medium containing higher amounts of xylose (28 g/L) and compared to a glucose-containing medium (28 g/L, control). These authors observed that after 111 h of fermentation the maximum biomass concentration was almost the same for the two carbon sources (4.14 and 4.30 g/L for glucose and xylose, respectively). However, the specific growth rate for glucose (0.1 h) was higher than that observed for xylose (0.05 h). Another study carried out with *R. toruloides* tested a higher xylose concentration than our study (40 g/L) and the authors also reported that the growth rate in glucose-containing medium was higher (twice) than for xylose medium, the exponential phase with glucose lasting 72 h as compared to xylose (40 h) [24]. The high contents of mannose found demonstrates the great value of açaí seeds as a potential culture medium for similar yeasts, which was confirmed by Byrtusová et al. [25], who demonstrated the influence of mannose on yeast growth as similar to the influence of glucose, the most assimilable carbon source by yeasts, to perform growth.

Regarding the potential microbial inhibitors produced during the acid hydrolysis of carbohydrates in the hydrolyzed liquor of the dried açai seeds (Table 2), acetic acid was the major detected compound (1.8 g/L), followed by hydroxymethylfurfural (338 mg/L), and furfural (10 mg/L), and a significant decrease (≈29.5%) in the contents of hydroxymethylfurfural was observed after the liquor neutralization. Furfural and hydroxymethylfurfural are derived from pentoses and hexoses degradation, respectively, while acetic acid is formed from the acetic groups present in the hemicellulosic fraction generated during steam explosion; they have been reported as inhibitor for the growth of several microorganisms by prolongation of the lag phase during fermentation [5,17]. However, the inhibitory concentration ranges of each compound should be systematically investigated, particularly for each interested microorganism to establish the allowed limits in the culture media to enable the use of a great variety of substrates, especially those that are low-cost and derived from agroindustrial wastes.

Lin et al. [26] reported an inhibitory effect on *R. glutinis* when acetic acid concentration reached 12 g/L, and furfural at 800 mg/L also caused the inhibition of yeast growth for 12 h. In contrast, hydroxymethylfurfural at 200 mg/L had no inhibitory effect on yeast growth, but at 400 mg/L, an inhibitory effect on biomass was observed. In another study, Vajzovic et al. [27] reported that furfural at concentrations lower than 3 g/L showed no inhibitory effect on the growth of *R. mucilaginosa*, and that xylose metabolism was negatively affected only from this value onwards.

### 2.5. Scanning Electron Microscopy (SEM) of the Dried Açai Seeds before and after Acid Hydrolysis Treatment with H_2_SO_4_

**Figure 5:**
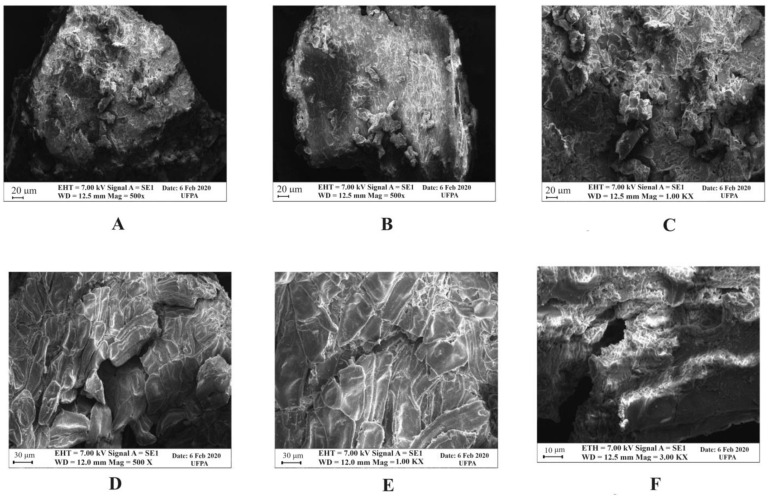
Micrographs of biomass of dried açai seeds before ((**A**) = 500× (**B**) = 500×; (**C**) = 1000×) and after the acid treatment ((**D**) = 500× (**E**) = 1000×; (**F**) = 3000×) with diluted H_2_SO_4_ at the optimal hydrolysis conditions (3.5% acid concentration (*w*/*v*), 25% solids concentration (*w*/*v*), during 70 min).

Through the micrographs obtained by SEM (Figure 5), it was possible to observe that the biomass of dried açai seeds before the acid treatment (Figure 5A–C) showed no damage or cracks in its fiber structures, with small irregular protuberances due to the milling process of the fruit. However, after the acid treatment with diluted H_2_SO_4_ (Figure 5D–F), it was possible to observe the loss of mass density, cracks, and accentuated cell disruption, promoting partial structural disorganization, making the material more porous and amorphous. These modifications are the result of the acid-solubilized mass (mainly hemicellulose) and partial modification of cellulose crystallization. According to the literature, the cracks and ruptures observed in the micrographs of the hydrolyzed açai seeds may be due to the breaking of bonds between carbohydrates and lignin [28].

## 3. Materials and Methods

### 3.1. Açai Seeds

The açai seeds (*Euterpe oleracea* Mart.) (25 kg) were donated by a fruit pulp processing Company, whose fruits were grown in Igarapé-Miri city, Pará State, Brazil (latitude 1°58′37″ S, longitude 48°57′34″ W). The access to açai fruits was registered in the Brazilian National System for the Management of Genetic Heritage and Associated Traditional Knowledge (SisGen AF18EDC). After removing the pulp and rind to produce açai juice, the açai seeds (25 kg) were collected at the company and directed to the laboratory. The açai seeds were washed with running water to remove solid wastes and other impurities, followed by sanitization by immersion in a sodium hypochlorite solution (100 mg/L) for 10 min. The seeds were dried at 65 °C in an oven with forced air circulation for 48 h, until moisture was below 10%, as monitored by an infrared moisture analyzer, according to the National Renewable Energy Laboratory [12]. The dried açai seeds were ground in a knife mill, subjected to sieving at 20 mesh (0.841 mm), and stored under vacuum in polypropylene plastic bags at −20 °C until use.

### 3.2. Characterization of Dried Açai Seeds

The contents of total proteins (conversion factor 6.25 from total nitrogen), total lipids, total acidity (expressed in meq NaOH/100 g), ashes, and pH values were determined according to the Association of Official Analytical Chemists [29]. The total carbohydrate contents (%) were calculated by the difference between 100 and the sum of the percentages of moisture, proteins, lipids, and ashes. The contents of total extractives (secondary metabolite compounds of vegetables that may include waxes, fats, resins, tannins, gums, sugars, starches, and pigments) were determined by gravimetry according to Sluiter et al. [30] to eliminate the usual overestimation of structural carbohydrates. Briefly, 4 g of dried açai seeds were rinsed in reflux with ethanol (99%) until complete decolorization of the solvent, followed by washing with distilled water and drying in an oven until dryness. All the results, in triplicate, were expressed in g/100 g (%).

### 3.3. Determination of Structural Carbohydrates

The structural carbohydrates were determined in the dried açai seed samples, after removing the extractives, followed by acid hydrolysis with H_2_SO_4_ 72%, according to Sluiter et al. [31]. The contents of structural carbohydrates were calculated after the determination of furfural, hydroxymethylfurfural, total lignin, monomeric sugars, and acetic acid.

#### 3.3.1. Determination of Furfural and Hydroxymethylfurfural Content

The furfural and hydroxymethylfurfural (HMF) contents were determined in a HPLC (Agilent HPLC, model 1260 Infinity, Santa Clara, CA, USA), equipped with a quaternary pump (G1311C), a Rheodyne injection valve with a 20 μL loop, an oven (G1316A), and a diode array detector (DAD) (G1328C). For all the chromatographic analyses, samples and solvents were filtered using 0.22 and 0.45 μm membranes, respectively (Millipore, Billerica, MA, USA). The compounds were separated in a C_18_ column (Nova-Pak Waters, Milford, MA, USA) with an isocratic mobile phase of acetonitrile/water (1:8, *v*/*v*) with 1% acetic acid, at 30 °C, flow rate at 0.8 mL/min, and the chromatograms were monitored at 280 nm for 20 min [3]. The furfural and HMF contents were determined, in triplicate, by analytical curves (0.001 to 0.1 g/L, R^2^ > 0.99), and the results are expressed in g/L.

#### 3.3.2. Determination of Total Lignin

The content of soluble and insoluble lignin (Lignin of Klason) was determined according to Sluiter et al. [31] Since part of the insoluble material of the dried açai seeds is composed of minerals (ashes), which are not soluble in acid, the ashes contents were also determined to avoid the overestimation of insoluble lignin values. The content of soluble lignin was determined by spectrophotometry at 280 nm. All the results, in triplicate, are expressed in g/100 g (%).

#### 3.3.3. Determination of Monomeric Sugars and Acetic Acid

The contents of cellobiose, glucose, mannose, xylose, and acetic acid in the hydrolysates were determined by HPLC (Thermo Fisher Scientific, SRVYER model, San Jose, CA, USA), coupled to a refractive index detector (SRVYER-RI), according to Martins et al. [3] The compounds were separated in an ion exchange column (Aminex HPX-87H) at 30 °C with a solution of 5 mM H_2_SO_4_ in deionized water as mobile phase at 0.6 mL/min (isocratic mode). The chromatographic separation was monitored by a refractive index detector (RID) at 35 °C for 20 min. The contents (g/L, *n* = 3) of each sugar and acetic acid were determined by external standardization with 6-point analytical curves ranging from 0.1 to 0.6 g/L (r^2^ > 0.99) with d-(+)-cellobiose, d-glucose, d-mannose, d-xylose, and acetic acid standards.

#### 3.3.4. Calculation of Structural Carbohydrates Contents

Cellobiose and glucose contents were converted into cellulose, using the conversion factors of 0.95 and 0.90, respectively, and mannose and xylose contents were converted into hemicellulose, using the conversion factor of 0.88. For the acetyl groups, the conversion factor from acetic acid to acetate of 0.717 was used, and for the calculation of the masses of furfural and HMF, the conversion factors of 1.375 and 1.286, respectively, were used, with furfural being converted to hemicellulose and HMF for cellulose. The conversion values are part of the known stoichiometry involved in the reaction, involving their respective molecular masses during the conversions, according to Sluiter et al. [32].

### 3.4. Determination of the Optimal Conditions for the Acid Hydrolysis of Dried Açai Seeds

The optimal conditions for the acid hydrolysis of the dried açai seeds as pre-treatment were determined, in a first moment, for two diluted acids (H_2_SO_4_ and H_3_PO_4_) using a central composite rotational design (CCRD) for each acid in order to select the most efficient acid in generating total reducing sugars. Both the CCRDs consisted of a 2^3^ factorial design plus 6 axial points and 3 repetitions at the central point, totaling 17 experiments for each diluted acid, with three independent variables at two levels (−1, +1): acid concentration (1 to 4%, *w*/*v*), hydrolysis time (30 to 90 min), and solids concentration (5 to 20%, *w*/*v*) (Table 3), for which the selected ranges were based on the results obtained by previous studies [3,6].

The response variables for the experimental designs using H_2_SO_4_ or H_3_PO_4_ were the content of total reducing sugars (TRS) and the combined severity factor of the acid treatment (CSF). The TRS contents were determined by spectrophotometry at 540 nm, according to the dinitro-3,5-salicylic acid (DNS) method [33], the contents (g/L, in triplicate) being obtained by external analytical curves with 10 points of D-glucose standard ranging from 0.1 to 1 g/L (R^2^ > 0.99). The CSF was calculated according to Equation (1).

(1) $$log(R_{0})=log\cdot \left[t\cdot exp\left(\frac{T-T_{ref}}{14.75}\right)\right]-\mathrm{pH}$$

where: *t* is the reaction time (min), *T* is the reaction temperature (°C), *T_ref_* is the reference temperature (100 °C), pH is the dilute acid solution; 14.75 is an arbitrary constant (ω) based on the activation energy of the reaction when assuming pseudo-first order kinetics.

In a second step, after selecting the most efficient acid that produced liquors with the highest yields of TRS to continuous the investigation, we also determined the levels of hydroxymethylfurfural (HMF) and solubilized mass yields (SM) for the same CCRD. The HMF contents were determined by spectrophotometry at 284 and 336 nm, according to the methodology of AOAC [29], and the percentages of SM of the solid fraction of the hydrolyzed core were determined according Equation (2).

(2) $$\mathrm{SM}\left(\%\right)=\left(\frac{m_{final}}{m_{initial}}\times 100\right)-100$$

where *m_initial_* is the initial dry mass of lignocellulosic material; *m_final_* is the final dry mass of lignocellulosic material.

In all the experiments of the acid hydrolysis, the ground dry açai seeds were weighed, transferred to borosilicate flasks, followed by the different acid and solid concentrations, and submitted to heating in an autoclave at 121 °C at different times, according to the selected CCRD experiment. After the acid hydrolysis step, the solid biomass was separated from the liquor by vacuum-filtration on qualitative filter paper (with mass previously known). The obtained liquor was directed for the TRS and HMF determinations by spectrophotometry during the CCRD experiments; and after establishing the optimal hydrolysis conditions, the newly hydrolyzed liquor was fully characterized by HPLC for the contents of cellobiose, glucose, xylose, arabinose, acetic acid, furfural, and HMF. In another set of experiments, the full characterization was also carried out for the newly hydrolyzed liquors produced at the optimal conditions and submitted to neutralization with K_2_SO_4_, autoclaved at 121 °C for 15 min, and filtered, according to common practices to be further used in fermentation processes. The solid biomasses obtained during vacuum-filtration were washed with distilled water until the washing water reached pH close to neutrality (pH 6.0–7.0). The biomass was dried in an oven at 105 °C with forced air circulation until constant mass for the SM (%) determination.

### 3.5. Scanning Electron Microscopy (SEM)

The ground dried açai seeds (in natura) and the dried biomass obtained after the pre-treatment with the selected acid, at the optimal hydrolysis conditions, were analyzed by SEM (Zeiss, EVO MA10, Germany) to observe the surface changes resulting from the pre-treatment. The samples were fixed with carbon tape on an aluminum support, then submitted to a 10 nm gold metallic coating in a metallizer. The operating conditions were electron beam current = 100 μA, constant accelerating voltage = 10 kV, and working distance = 8.5 mm.

### 3.6. Statistical Analysis

The results (mean ± standard deviation, *n* = 3) were analyzed by the Statistica 7.0 software (Statsoft Inc., Tulsa, OK, USA) through analysis of variance (ANOVA), Tukey’s test, and Student’s *t*-test (*p* < 0.05). All data obtained by the CCRD experiments were fitted to second order polynomial models to determine the optimal hydrolysis conditions by the response surface methodology, and Pareto charts were built at 10% of statistical significance (*p* ≤ 0.1) to investigate the effects of acid hydrolysis on the studied variables. The adequacy to the second order models was determined by evaluating the coefficient determination (R^2^), lack of fit, and Fisher test values (F-value) through ANOVA at 10% of statistical significance (α = 0.1). The obtained mathematical models were used to generate response surfaces and contour plots to provide the optimal acid hydrolysis conditions to obtain a liquor with high contents of simple carbohydrates and low levels of potential microbial inhibitors.

## 4. Conclusions

Pre-treatment with diluted H_2_SO_4_ was more efficient than H_3_PO_4_. The optimal hydrolysis condition established for the açai seeds for fermentation processes provided a concentration of 55 g/L of sugars, consisting mainly of mannose (93%) and low levels of inhibitors. Thus, açai seeds can be seen as a promising byproduct with both high value and high potential to be used as a substrate in biotechnological processes, contributing to an environmental and socioeconomic strategy for the use of agroindustrial waste.

## Figures and Tables

**Table 1:** Combined severity factor (CSF), contents of total reducing sugars (TRS), hydroxymethylfurfural (HMF) and solubilized mass obtained during the central composite rotational design (CCRD) runs for the acid hydrolysis of açai seeds using sulfuric acid (H_2_SO_4_).

	Level of Independent Variables				Dependent Variables		
Runs	X_1_	X_2_	X_3_	CSF	TRS (g/L)	HMF (mg/L)	Solubilized Mass (%)
1	1 (−1)	30 (−1)	5 (−1)	1.06	12.25	5.60	27.93
2	4 (+1)	30 (−1)	5 (−1)	1.21	17.65	33.68	38.58
3	1 (−1)	90 (+1)	5 (−1)	1.53	14.76	12.82	31.94
4	4 (+1)	90 (+1)	5 (−1)	1.68	25.40	90.30	48.83
5	1 (−1)	30 (−1)	20 (+1)	1.06	28.98	19.92	21.53
6	4 (+1)	30 (−1)	20 (+1)	1.21	59.60	82.06	35.23
7	1 (−1)	90 (+1)	20 (+1)	1.53	57.54	14.79	29.25
8	4 (+1)	90 (+1)	20 (+1)	1.68	103.12	448.83	48.49
9	0 (−1.68)	60 (0)	12.5 (0)	NC1	6.93	1.49	11.56
10	5 (+1.68)	60 (0)	12.5 (0)	1.62	44.70	148.90	49.58
11	2.5 (0)	9.54 (−1.68)	12.5 (0)	0.69	15.93	<LOD	23.52
12	2.5 (0)	110 (+1.68)	12.5 (0)	1.75	51.34	191.34	41.22
13	2.5 (0)	60 (0)	0.1 (−1.68)	1.49	<LOD	0.59	NC2
14	2.5 (0)	60 (0)	25 (+1.68)	1.49	63.95	87.01	33.28
15	2.5 (0)	60 (0)	12.5 (0)	1.49	49.06	70.05	37.13
16	2.5 (0)	60 (0)	12.5 (0)	1.49	48.16	70.58	38.50
17	2.5 (0)	60 (0)	12.5 (0)	1.49	48.92	72.53	37.18

X_1_: Acid concentration (%, *w*/*v*). X_2_: Hydrolysis time (min). X_3_: Solids concentration (%, *w*/*v*). <LOD: below the limit of detection (equal 0 for the prediction purpose). NC1: Combined severity factor not calculated given the 0% diluted acid concentration. NC2: Not calculated (equals to 0) due to the low solid concentration (0.1%, *w*/*v*).

**Table 2:** Contents of sugars and microbiological inhibitors in the newly hydrolyzed liquor and after neutralization both produced after acid hydrolysis of dried açai seeds with H_2_SO_4_ at the optimal conditions.

	Hydrolyzed Liquor	
	Newly Hydrolyzed	After Neutralization *
**Sugars**		
Mannose (g/L)	51 ± 1 ^a^	42.1 ± 0.1 ^b^
Xylose (g/L)	1.9 ± 0.2 ^a^	1.4 ± 0.2 ^b^
Glucose (g/L)	1.3 ± 0.1 ^a^	1.0 ± 0.1 ^a^
Cellobiose (g/L)	0.9 ± 0.1 ^a^	0.7 ± 0.1 ^a^
Sum of the sugars (g/L)	55 ± 1 ^a^	45.2 ± 0.4 ^b^
**Microbial inhibitors**		
Hydroxymethylfurfural (mg/L)	338 ± 50 ^a^	238 ± 20 ^b^
Furfural (mg/L)	10 ± 1 ^a^	9 ± 1 ^a^
Acetic acid (g/L)	1.8 ± 0.1^a^	1.76 ± 0.04 ^a^

* Liquor hydrolyzed under the optimal acid hydrolysis conditions, followed by neutralization with K_2_SO_4_, autoclaved at 121 °C for 15 min and filtered, according to common practices used in fermentation processes. The values (mean ± standard deviation) with the same superscript letters in the same line are not statistically different (*p* > 0.05) (Student’s *t*-test).

**Table 3:** Range of the independent variables selected for the central composite rotational design (CCRD) during the determination of the optimal conditions for the acid hydrolysis of dried açai seeds using H_2_SO_4_ and H_3_PO_4_ as pre-treatment.

Independent Variable		Level				
		−1.68	−1	0	+1	+1.68
Acid concentration (%, *w*/*v*)	X_1_	0	1	2.5	4	5
Hydrolysis time (min)	X_2_	9.5	30	60	90	110
Solids concentration (%, *w*/*v*)	X_3_	0.1	5	12.5	20	25

## Data Availability

All data generated or analyzed during this study are included in this published article and its Supplementary Information file.

## Supplementary Materials

The following supporting information can be downloaded at: https://www.mdpi.com/article/10.3390/molecules28186661/s1, Supplementary Table S1: Total reducing sugars (TRS) and combined severity factor (CSF) obtained during the central composite rotational design (DCCR) runs for the acid hydrolysis of açai seeds using phosphoric acid (H_3_PO_4_). Supplementary Table S2—*t* and *p* value at 90% confidence for the studied parameters in the experimental design. Supplementary Table S3—ANOVA (10% significance) for the adequacy of the quadratic response surface models to determine the contents of total reducing sugars (TRS), hydroxymethylfurfural (HMF) and solubilized mass (SM) after acid hydrolysis of dried açai seeds. Supplementary Figure S1—Profiles for the predicted values and desirability function used to predict the contents of total reducing sugars (TRS), hydroxymethylfurfural (HMF), and solubilized mass at the chosen optimal condition for the acid hydrolysis of dried açai seeds: with 3.5% H_2_SO_4_ + 25% solids during 70 min in autoclave.
