# Supplementary material for: Açai Seeds (Euterpe oleracea Mart) Are Agroindustrial Waste with High Potential to Produce Low-Cost Substrates after Acid Hydrolysis
Source: Molecules. 2023 Sep 16;28(18):6661. doi: 10.3390/molecules28186661 (PMC10536898; doi:10.3390/molecules28186661)

Molecules Journal

SUPPLEMENTARY MATERIAL

Açai Seeds (*Euterpe oleracea* Mart) are Agroindustrial Wastes with High Potential to Produce Low-Cost Substrates after Acid Hydrolysis

Willen Silva Igreja[a], Luiza Helena da Silva Martins[a,b], Rafaela Rodrigues de Almeida[c], Johnatt Allan Rocha de Oliveira[a,d], Alessandra Santos Lopes[a,c], Renan Campos Chisté [a,c,]*

[a]*Graduate Program in Food Science and Technology, Institute of Technology (ITEC), Federal University of Pará (UFPA), 66075-110, Belém, Pará, Brazil.*

[b]*Instituto de Saúde e Produção Animal (ISPA), Universidade Federal Rural da Amazônia (UFRA), 66077-830, Belém, Pará, Brazil.*

[c]*School of Food Engineering (FEA), Institute of Technology (ITEC), Federal University of Pará (UFPA), 66075-110, Belém, Pará, Brazil.*

[d]*Nutrition College, Federal University of Pará (UFPA), Belem 66110-072, PA, Brazil.*

Luiza Helena da Silva Martins – ORCID 0000-0003-1911-4502

Johnatt Allan Rocha de Oliveira - ORCID 0000-0002-1778-3043

Alessandra Santos Lopes – ORCID 0000-0002-8584-5859

Renan Campos Chisté – ORCID 0000-0002-4549-3297

---

*Corresponding author: rcchiste@ufpa.br (Ph.D Renan Campos Chisté).
Faculdade de Engenharia de Alimentos (FEA), Instituto de Tecnologia (ITEC), Universidade Federal do Pará (UFPA). Rua Augusto Corrêa, 01, Guamá, CEP: 66075-110, Belém, Pará, Brasil.

Supplementary Table 1. Total reducing sugars (TRS) and Combined Severity Factor (CSF) obtained during the Central Composite Rotational Design (CCRD) runs for the acid hydrolysis of açaí seeds using phosporic acid (H_3PO_4).

Level of independent variables				Dependent variable	
				**H_3PO_4**	
**Runs**	X_1	X_2	X_3	**CSF**	**TSR (g/L)**
1	1 (-1)	30 (-1)	5 (-1)	0.96	2.93
2	4 (+1)	30 (-1)	5 (-1)	1.12	3.93
3	1 (-1)	90 (+1)	5 (-1)	1.43	4.59
4	4 (+1)	90 (+1)	5 (-1)	1.59	7.13
5	1 (-1)	30 (-1)	20 (+1)	0.96	11.92
6	4 (+1)	30 (-1)	20 (+1)	1.12	15.32
7	1 (-1)	90 (+1)	20 (+1)	1.43	22.13
8	4 (+1)	90 (+1)	20 (+1)	1.59	33.97
9	0 (-1.68)	60 (0)	12.5 (0)	NC	9.62
10	5 (+1.68)	60 (0)	12.5 (0)	1.48	30.07
11	2.5 (0)	9.54 (-1.68)	12.5 (0)	0.55	11.04
12	2.5 (0)	110 (+1.68)	12.5 (0)	1.61	22.77
13	2.5 (0)	60 (0)	0.1 (-1.68)	1.35	< LOD
14	2.5 (0)	60 (0)	25 (+1.68)	1.35	29.51
15	2.5 (0)	60 (0)	12.5 (0)	1.35	20.00
16	2.5 (0)	60 (0)	12.5 (0)	1.35	22.19
17	2.5 (0)	60 (0)	12.5 (0)	1.35	21.29

X_1: Acid concentration (%, w/v). X_2: Hydrolysis time (min). X_3: Solids concentration (%, w/v). < LOD: below the limite of detection. NC: Combined Severity factor not calculated given the 0% diluted acid concentration.

Supplementary Table 2. t and p value at 90% confidence for the studied parameters in the experimental design.

Factor	t value (TRS)	p value (TRS)	t value (HMF)	p value (HMF)	t value (Solubilized mas)	p value (Solubilized mas)
**Mean/Interaction**	171.6399	0.000034	92.8866	0.000116	82.2134	0.000148
**X_1 (Linear)**	87.2050	0.000131	176.2357	0.000032	43.2951	0.000533
**X_1 (Quadratic)**	-39.2657	0.000648	13.7007	0.005285	**-1.2569**	**0.335682**
**X_2 (Linear)**	79.0235	0.000160	155.0748	0.000042	22.6748	0.001939
**X_2(Quadratic)**	-19.8918	0.002518	32.6532	0.000937	**1.4774**	**0.277625**
**X_3(Linear)**	160.2199	0.000039	118.1064	0.000072	14.9800	0.004427
**X_3(Quadratic)**	-24.3874	0.001677	-15.4188	0.004180	-22.5319	0.001964
**X_1 by X_2**	14.9660	0.004435	114.0549	0.000077	5.3606	0.033082
**X_1 by X_3**	44.1388	0.000513	105.7494	0.000089	2.4573	0.133285
**X2 by X3**	44.9127	0.000495	80.6162	0.000154	3.0580	0.092360

Supplementary Table 3 – ANOVA (10% significance) for the adequacy of the quadratic response surface models to determine the contents of total reducing sugars (TRS), hydroxymethylfurfural (HMF) and solubilized mass (SM) after acid hydrolysis of dried açai seeds with H_2SO_4.

**Variation source**	**Sum of squares**	**Degrees of freedom**	**Mean Square**	**F_{value}**	**R^2**
**TRS (g/L) = $47.83+11.47X_1-5.73X_1^2+10.37X_2-2.88X_2^2+21.10X_3-3.58X_3^2+2.56X_1X_2+7.55X_1X_3+7.69X_2X_3$**					
Regression	10659.8	9	1184.4	20.11	0.96
Residue	412.2	7	58.9		
Lack of fit	411.71	5	82.343	351.09	
Pure error	0.47	2	0.235		
Total	11072.00	16			
**HMF (g/L) = $69.81+62.51X_1+5.39X_1^2 + 54.90X_2+12.75X_2^2+ 41.96X_3-6.11X_3^2+52.66X_1X_2+48.33X_1X_3+37.22X_2X_3$**					
Regression	173051.3	9	19227.70	6.92	0.89
Residue	19452.5	7	2779.07		
Lack of fit	19449.1	5	9.8863	2280.57	
Pure error	3.4	2	2.9883		
Total	192503.8	16			
**SM (%) = $36.08+9.14X_1 + 4.77X_2+3.17X_3-5.32X_3^2+1.47X_1X_2+0.84X_2X_3$**					
Regression	1955.5	9	217.27	1.93	0.71
Residue	786.4	7	112.34		
Lack of fit	785.2	5	157.0	260.15	
Pure error	1.207	2	0.604		
Total	2741.8	16			

$F_{0.1;\,9;\,7} = 2.72$; $F_{0.1;\,5;\,2} = 9.29$ (F = Tabulated value from Fisher test). The mathematical models were considered significant ($p \leq 0.1$) when the regression F_{values} were three times higher than $F_{tabulated}$ and as predicted when the lack of fit F_{values} were lower than the $F_{tabulated}$ value.

Supplementary Figure 1 – Profiles for the predicted values and desirability function used to predict the contents of total reducing sugars (TRS), hydroxymethyl furfural (HMF) and solubilized mass at the chosen best condition for the acid hydrolysis of dried açai seeds: with 3.5% H_2SO_4 + 25% solids during 70 min in autoclave.

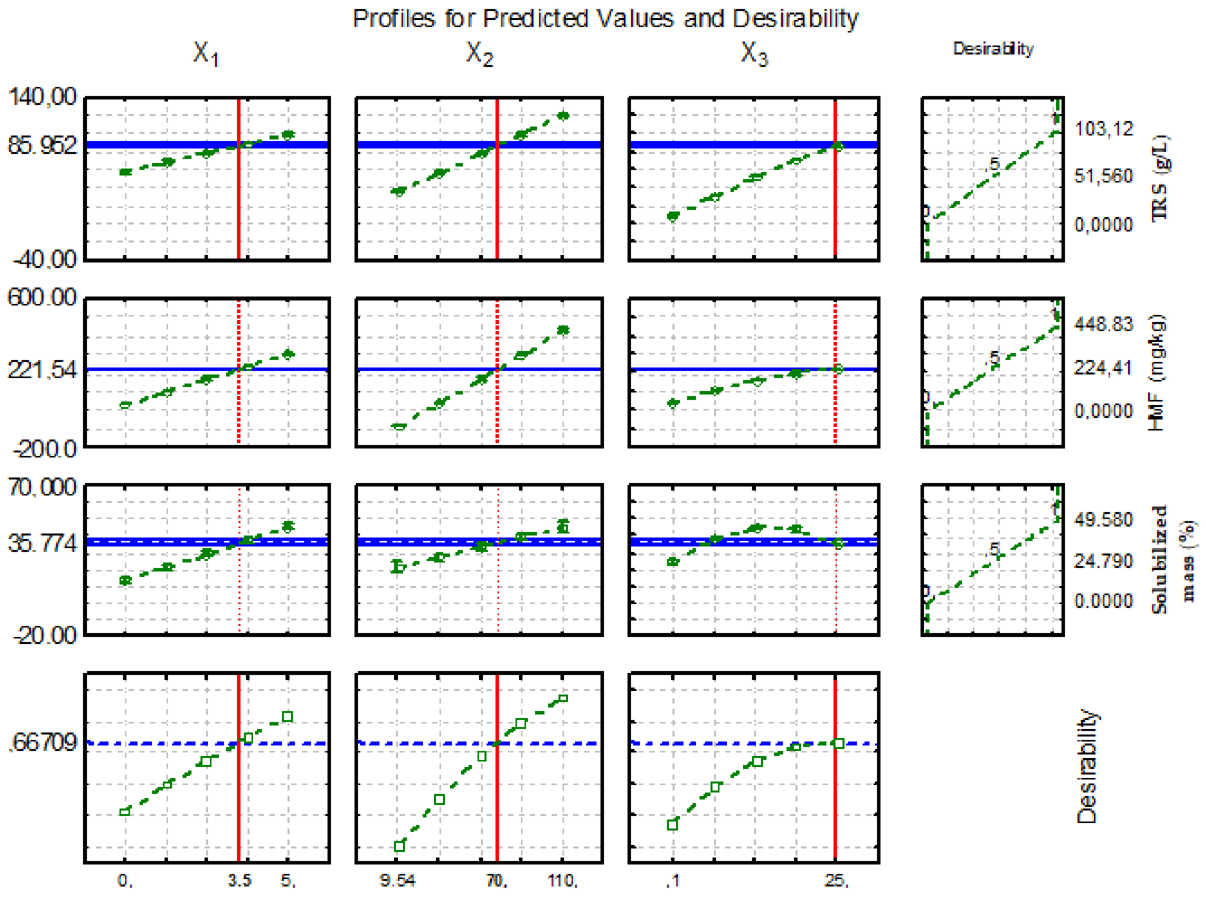

Supplement: Supplementary file 1 [file molecules-28-06661-s001.zip › molecules-2587296-supplementary.pdf]
